# HODet:Horizontal-to-Oriented Object Detection with simplified annotation

**DOI:** 10.1038/s41598-026-58703-8

**Published:** 2026-08-03

**Authors:** Jinlin Chen, Yiquan Wu, Yubin Yuan

**Affiliations:** 1 College of Electronic and Information Engineering, Nanjing University of Aeronautics and Astronautics, Nanjing, China; 2 College of Finance and Mathematics, Huainan Normal University, Nanjing, China

**Keywords:** Machine learning, Oriented object detection, Loss function, Residual network, Drone vision, Engineering, Mathematics and computing

## Abstract

The recognition and positioning of characters on the water gauge are important components of artificial intelligence system for reading ship draft weighing. Meanwhile, the manual annotation of oriented objects has a large workload and low accuracy. To address these issues, this paper proposes a novel oriented detection framework. It introduces three additional parameters to refine the horizontally detected bounding boxes from the model and employs two symmetric functions to confine the angles and scales within specified ranges. In the training and testing of the model, only the horizontal annotation information of the object is needed, reducing the workload of annotation of the target in the dataset. A residual network has been added to the backbone of object detector(YOLO) to enhance the feature extraction capability of deep modules and improve the sensitivity to small objects. For more accurate oriented estimation, we utilize improved Intersection over Union(IoU) to the bounding box regression loss.This method is particularly effective for objects with a dominant orientation and approximately rectangular shapes, such as the characters, vehicles, and buildings commonly found in aerial imagery. A simple implementation of our method has achieved state-of-the-art performances on aerial objects datasets, with a negligible reduction to detection speed. After applying the novel oriented detection method to the intelligent system, real-time testing was conducted on 120 drone videos, resulting in a 35.3% improvement in the accuracy of the system’s water gauge readings.

## Introduction

**Fig. 1:**
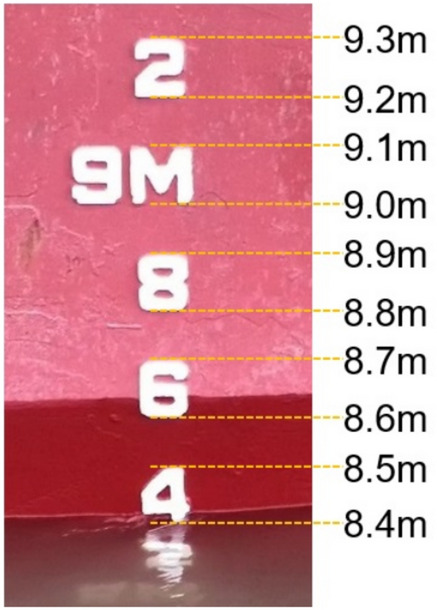
Schematic diagram of metric system for ship water gauge.

Artificial intelligence technology has been widely applied in many fields such as remote sensing monitoring^1^, vehicle tracking^2^, maritime shipping, and disaster rescue, demonstrating strong potential. In the bulk cargo transportation industry, the intelligent ship draft weighing method^3^represents one of the important application prospects of this technology. Usually, the water gauge of a ship is located on the left and right sides of the bow, mid-ship, and stern of the ship, with a total of six water gauge markings. On the hull, each water gauge is arranged in a straight line, and some ships also adopt a segmented arrangement. The ship water gauges rely on two types: metric and imperial^4^. The metric characters are Arabic numerals with a unit of cm, and the height and spacing between adjacent characters are 10 cm, as shown in Fig.1. The imperial characters are Roman numerals, with the unit of ’in’, and the height and spacing between adjacent characters are 1 inch. At present, the main problems with manually reading ship water gauges are high cost, numerous disputes, difficulty in real-time reading, and so on^5^. To address these issues in reading water gauge, we have designed a novel water gauge reading system(WGRS) based on unmanned aerial vehicle (UAV) which consists of four modules: water line extraction, water gauge character recognition, water gauge modeling, water gauge calculation. Despite the notable progress achieved by WGRS approaches, the limited accuracy in water gauge character(WGC) detection remains one of the primary sources of system errors.

However, there are many bottlenecks in the existing Deep Learning-based water gauge images in real applications: The WGC has corroded, rusted, or eroded due to long-term outdoor exposure, water immersion, or air pollution^6^; during the process of loading and unloading goods at the dock, the shaking of the ship’s hull, the fluctuation of water waves, and the tilting of the hull cause the WGC captured by the drone to be tilted or blurred^7^-^8^; the nighttime lighting, weather conditions, and work environment during loading and unloading of goods will affect the clarity of the character; and the accuracy of WGC detection will be affected by factors such as the flight stability and attitude of the drone, the angle of the aerial photography.

**Fig. 2:**
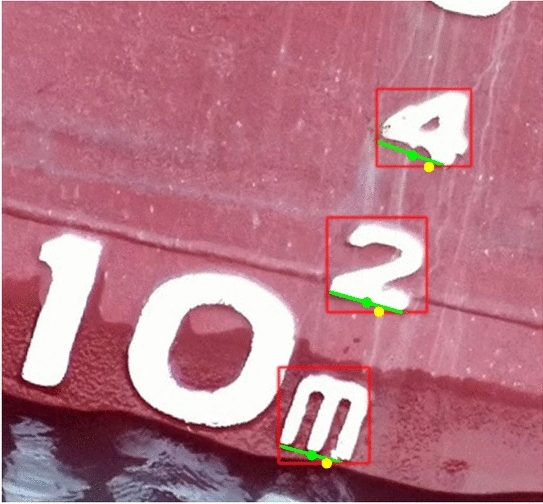
Bottom line of water gauge.

The manual reading of ship water gauges is based on the midpoint position of the lower edge of the WGC. However, the WGC detection model labels characters with a horizontal rectangular box(HRB). In this case, there may be deviations in the water gauge values read by the system with the same method as described above. As shown in the following Fig.2, in which significant deviation exist between the midpoint of the red horizontal rectangle and the green point under the character. For large vessels, this deviation may cause unnecessary commercial disputes. As a consequence, the objective of this paper is to harness Horizontal-to-Oriented Object Detection(HODet) to detect WGC as accurately as possible, thereby improving the accuracy of the system in reading water gauges.

In summary, our contributions are in three-fold:

- We propose a new method for detecting oriented-WGC. This object detector(YOLO) will predict the directional rectangular box parameters of WGC with four branches. To improve the small object detection capability of the model, a skip connection residual network was applied in the feature extraction part of YOLO, enhancing the deep network’s ability to extract features of WGC.

- This article describes the oriented detection of WGC as a multitasking problem, in which utilize the detection head generated by YOLO to predict character scores, HRB, and the rotation angles and scaling ratios of the two diagonals of the HRB, which is beneficial to complete the prediction of oriented rectangular box(ORB).

- The detection method calculate the loss function with improved MPDIoU^9^ to further enhances the accuracy of oriented detection of WGC.

It is noteworthy that the proposed geometric formulation is specifically designed for objects that can be well-approximated by rotated rectangles, which aligns perfectly with the characteristics of WGCs and many other man-made targets in remote sensing.

## Related work

Recent advances in oriented object detection have demonstrated significant importance across various industrial applications, including aerial imagery^10^-^11^,scene text recognition^12^, facial recognition^13^, and beyond. Numerous well-designed object detection methods^14^ have achieved promising results on large-scale datasets. However, given the prevalence of arbitrarily oriented targets in detection datasets, oriented object detection still faces notable challenges compared to traditional horizontal object detection^15^.

### Fully-supervised oriented object detection

Existing fully-supervised oriented detection methods can be broadly categorized into three groups: rotation-angle-based rectangular box prediction^16^, polygon prediction, and object feature coverage^17^.

In the rotation-angle-based rectangular box prediction approach, a detection model typically predicts at least four parameters to represent the HRB of the target, followed by additional output channels for rotation angle estimation. Representative methods in this category include Oriented R-CNN^18^, RoI Transformer^19^, Rotating RepPoint^20^, and Oriented YOLO^21^. Many of these methods regress the angle directly, which may encounter loss discontinuity^22^ and regression inconsistency^23^ due to the periodic nature of angular values.

The essential reason for the boundary problem lies in the discontinuity of the angle values of the orientation box. In earlier times, Raghunandan^24^ works by mapping the axis-aligned rectangle to feature map and dividing it into a *H×* grid of sub-windows, and then affine transformation is used to calculate the rotated position for each sub-window. Yang^25^ designs an angle correction module based on polar coordinate decomposition with adding to existing orientation detection methods. To solve the problem of boundary discontinuity, Jeffri^26^ represent a oriented object through a two-dimensional possibility rotated Gaussian distribution, abbreviated as Gaussian bounding boxes (GBB). But directly regressing GBB is susceptible to numerical instability. Zhou^27^ optimize GBB with linearly transforming, which avoids the boundary discontinuity problem and has high numerical stability. Fang^28^stacked multiple Vision Transformers blocks to form a Spatial Transform Decoupling (STD), and then predicted the center position, size, and angle of oriented boxes thought separate network branches. More recently, the authors in^29^ adopt a novel rotary angle encoder that employs an angle coordinate system and performs angle correction to mitigate boundary discontinuity and square-like problems. Wei^30^ extracts dense pseudo labels from the prediction graph of the teacher model and selected the predictions of the student model at the same position to obtain a series of pseudo label prediction pairs. Finally, the object-oriented detection task was completed by using adaptive weighting to adjust the consistency between each pseudo label and the predicted label. Yang^31^ applies a feature refinement module to achieve feature reconstruction of bounding boxes, further aligning the oriented target bounding boxes.

The polygon prediction method generates the oriented box of the object by predicting the position of the vertices of a quadrilateral, therefore, the detection model needs to predict at least 8 parameters^32^. The method in^33^ regresses 8 parameters of a quadrilateral to represent the four vertex positions of the oriented box. It is good at detecting polygonal targets but not fit for oriented objects. Xu^34^ achieves prediction of the center, length, and width of a horizontal rectangular box based on four parameters, and the other four parameters to predict the vertex offset of the rectangular box. Xie^18^proposes an instance based segmentation method that moves the four points of the horizontal border to form a parallelogram. This method only requires 6 parameters of HRB, namely center, length, width, and translation of two vertices.

The feature coverage is achieved by covering the object region with anchor points or small rectangular boxes to obtain oriented detection^35^. A set of points distributed within the mainly region of the object can provide finer grained positional representation, accordingly, RepPoint-based methods provide new alternatives for oriented object detection^36^. Zhou^37^ proposes to directly predict the oriented proposals with representative point learning, in view of the fact that points’ learning is more flexible and distribution is easier to describe the angle and size of rotating objects. However, it is worth noticing that low-quality anchor points cannot cover objects. As a consequence, well designed anchors with different angles, scales and aspect ratios are required, which however leads to massive computations and memory footprint. Han^17^ adaptively align the feature according to the corresponding anchor boxes with an Alignment Convolution. Li^20^ proposes an effective adaptive point learning and sample allocation scheme for selecting representative 9-point samples to cover objects. Additionally, Zhang^38^ cover the object with multiple small rectangular boxes. The method used local perceptual clustering algorithm to group dense detection boxes into different clusters, and then constructed an example graph for detection boxes belonging to one cluster to generate oriented rectangular boxes, but the inference time is longer at the expense of network parallelism.

### Weakly-supervised oriented object detection

Recent advances in weakly supervised oriented object detection reduce annotation costs while retaining accuracy. For example, a geometry-aware enhancement network^39^ learns geometric priors for remote sensing. Inspired by it, our HODet uses only horizontal boxes and a novel parameterization with two symmetric activation functions, achieving a better cost-precision trade-off. Compared with natural objects, printed texts are relatively regular, and using HODet to detect such text objects is feasible^33^. Consequently, research on text-based oriented object detection has produced many results^40^. For water gauge characters(WGC) in UAV images, datasets are crucial; however, oriented annotation is labor-intensive and often inaccurate, requiring expensive investments before model training. Therefore, starting from horizontal bounding boxes(HRB) and directly achieving oriented detection under weak supervision has become an important research direction.

Earlier works, such as Shivakumara^41^, combined axis-aligned bounding boxes with horizontal boxes to improve directional detection accuracy, but their accuracy depends on two boxes and affects computational speed. Yang^42^ proposed H2RBox using only horizontal box annotations for weakly-supervised training, with an improved version H2RBox-v2^43^. However, these methods require targets to have strong symmetry, which may not hold for water gauge characters that are often partially occluded, rusted, or eroded. The ABBSPO framework^44^ introduces adaptive bounding box scaling and a symmetric prior angle loss, but it still assumes certain geometric priors. Unlike these symmetry-dependent approaches, later weakly supervised methods have explored more flexible and annotation-efficient strategies.

For instance, Wholly-WOOD^45^leverages diverse label forms (points, HBoxes, RBoxes) in a unified framework, approaching RBox-trained performance, but its multi-stage training and mixed-label handling add complexity compared to our single-stage HODet. Similarly^46^, proposed a weakly supervised method for oriented ship detection in SAR images using multiscale feature enhancement and angle encoding, yet it is specifically designed for SAR imagery and requires careful tuning for optical UAV images^47^. presented WSODet, a weakly supervised oriented detector for aerial objects that uses layerwise relevance propagation and point set representation; however, its performance heavily depends on the quality of initial proposals.

Other efforts have explored even weaker forms of supervision^48^. introduced explicit and implicit box equivariance learning for weakly-supervised rotated object detection, achieving good results but requiring careful design of equivariance constraints. Pointbased supervision has also attracted attention^49^: proposed a point-based weakly semi-supervised method for vehicle detection in optical remote sensing images, reducing annotation cost to single points per object^50^. presented a relational matching approach for weakly semi-supervised oriented object detection, leveraging relationships among unlabeled data^51^. proposed a weakly semi-supervised method with only point annotations, combining self-training with point supervision. Such methods drastically reduce annotation cost but rely on additional pipelines or complex matching strategies, and their accuracy on tiny, densely arranged characters like WGC remains unverified.

Although current approaches have made good progress, they are still not suitable for directional detection of WGC, as the production of oriented datasets for water gauge characters remains difficult. In the following content, we propose a new method for directional detection that balances detection accuracy while saving the cost of oriented data production. Our HODet framework uniquely combines (1) a lightweight 7-parameter oriented box representation with symmetric activation functions, (2) improved residual connections for small object feature retention, and (3) an enhanced MPDIoU loss for accurate regression, achieving state-of-the-art results on both public remote sensing datasets and our real-world water gauge dataset.

## Proposed approach

The object detection network uses YOLOv8, which was proposed in 2023. YOLO’s idea is to directly regress the target position and predict the category in the input image, which makes YOLO have faster detection speed. This design builds upon the broad academic consensus regarding YOLOv8’s community support, computational efficiency, and technical reliability, thereby ensuring the robustness of our research baseline^52^.

### Optimization of feature extraction network

**Fig. 3:**
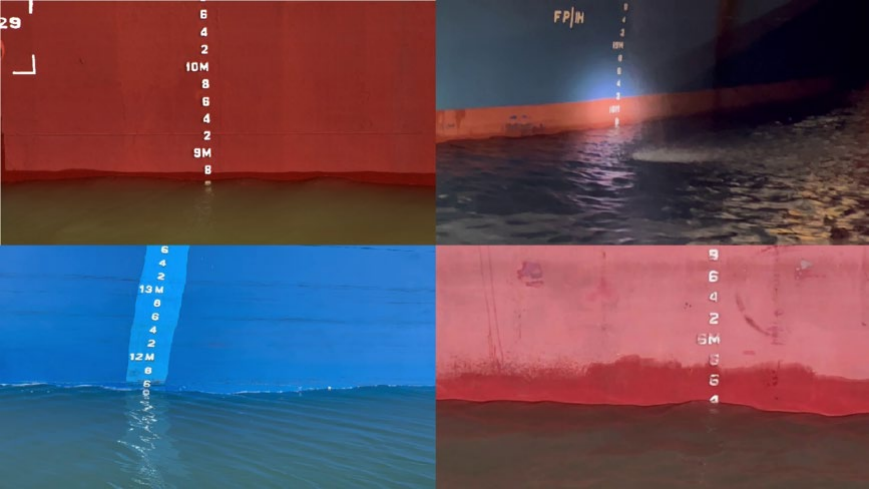
Ship water gauge image from UAV perspective.

**Fig. 4:**
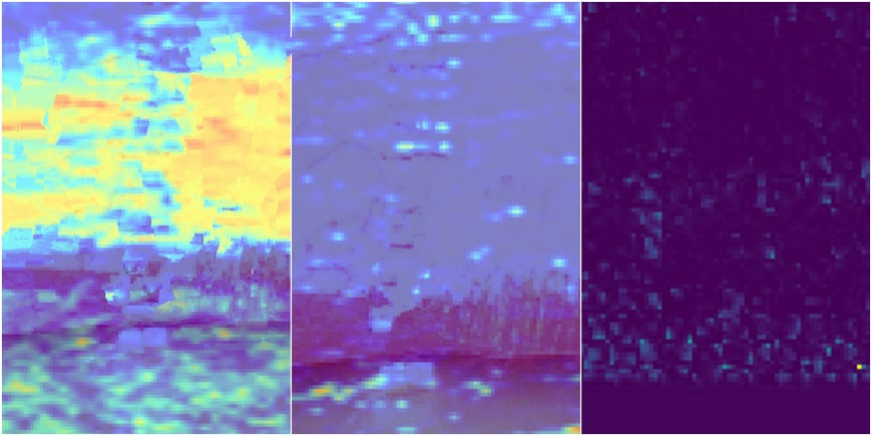
Visualization of Channel 1 feature maps for YOLOv8’s 2nd, 3rd, and 4th C2f layer features.

When capturing videos of ship hulls, the drone’s camera is typically not positioned too close to the hull. Consequently, the WGC in the image appear very small. The background usually consists of the ship’s hull and the water surface, presenting a relatively monotonous color palette, as illustrated in Fig.3. Deeper networks are more prone to losing original data features^53^. As a result, deep neural networks often face issues such as slow convergence or suboptimal convergence performance, a challenge also present in our water gauge detection model. When such water gauge images are input into the YOLOv8 detection model, the increasing number of network layers enlarges the receptive field, allowing the model to extract more global information. Feature map visualizations of a sample water gauge image from the shallow, middle, and deep C2f modules of the model are shown in Fig. 4. It can be observed that the visualization feature map from the deeper C2f module contains relatively less WGC information. This phenomenon is attributed to the extensive background in the original image overwhelming the character information of the water gauge, resulting in the model extracting very limited character object information. For this type of imagery, the deep C2f modules in the YOLOv8 detection model do not perform adequately. Therefore, further optimization of the model’s feature extraction module is necessary to enhance the capability of YOLOv8’s deep network in extracting object information.

While skip connections in traditional residual networks treat features from different layers equally, such uniform fusion may not be optimal for tasks requiring fine-grained detail preservation, such as small character detection in water gauge images. Inspired by adaptive feature fusion mechanisms like BiFPN^54^ and attention-based weighting, we introduce a learnable weighted summation of multi-level features to enhance information retention across network depths. This improvement is motivated by the fundamental role of residual connections in mitigating gradient vanishing and enabling deeper networks to improve accuracy through increased depth, as established in prior work^55^. However, under network degradation scenarios where shallower networks outperform deeper ones, simple feature propagation may still fall short in preserving crucial low-level details.

**Fig. 5:**
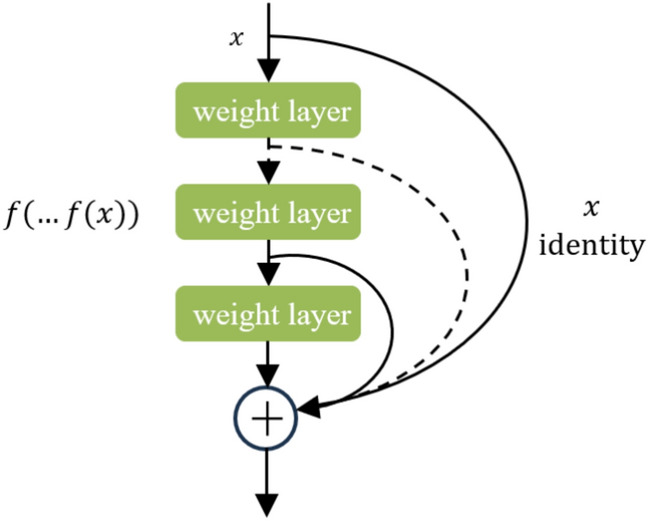
Improved residual network.

In our implementation within the backbone, we integrate shallow C2f features into deeper C2f modules via adaptively weighted skip connections. As illustrated in Fig. 5, the improved residual network learns residual mappings from the input *x*, and the overall output of a deep block can be expressed as a weighted sum of the outputs of multiple residual blocks at different depths:

1 $$\begin{aligned} H_n(x) = \alpha _n f^{(n)}(x) + \alpha _{n-1} f^{(n-1)}(x) + \dots + \alpha _1 f(x) + \alpha _0, \end{aligned}$$

where $f^{(k)}(x)$ denotes the output after applying *k* consecutive residual blocks, and $\alpha _i \ge 0$ are learnable weighting factors satisfying $\sum _{i=0}^{n} \alpha _i = 1$. These weights are optimized via backpropagation and initialized as uniform values ($\alpha _i = 1/(n+1)$) to enable equal contribution at the start of training.

This design is motivated by the observation that shallow features contain fine-grained edge and texture information critical for small object localization, whereas deep features encode semantic context but often lose spatial details(Fig. 4). By allowing the network to adaptively prioritize shallow residuals ($\alpha _0,\alpha _1$) or deep residuals ($\alpha _n$) per training sample, the model dynamically balances detail preservation and semantic abstraction. From a gradient flow perspective, the total gradient of the loss $\mathcal {L}$ with respect to the input *x* becomes:

2 $$\begin{aligned} \frac{\partial \mathcal {L}}{\partial x} = \sum _{i=0}^{n} \alpha _i \cdot \frac{\partial \mathcal {L}}{\partial H_i} \cdot \frac{\partial H_i}{\partial x}, \end{aligned}$$

where $H_i$ denotes the *i*-th residual mapping. Unlike standard residual networks where all residuals are summed with equal weight (implicitly $\alpha _i=1$), the learnable weights provide a flexible gating mechanism that prevents the vanishing of low-level gradient signals in deep layers. This is particularly beneficial for water gauge characters, which occupy only 10–30 pixels in height and are easily overwhelmed by homogeneous background. The weighted residual connections ensure that shallow, high-resolution features survive to deeper C2f modules, effectively increasing the signal-to-noise ratio for small target features.

**Fig. 6:**
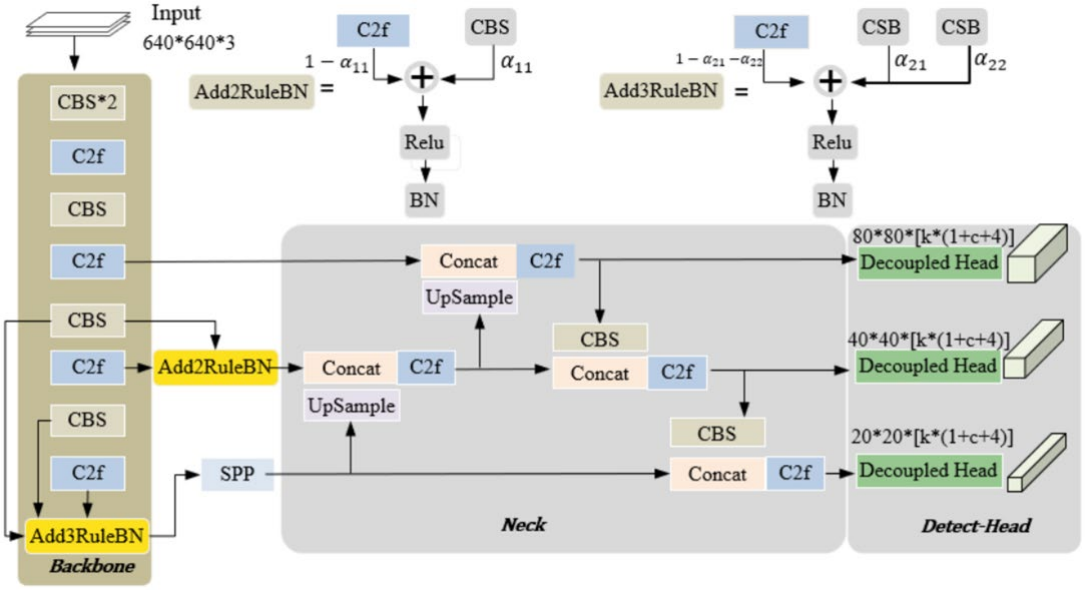
Backbone of YOLOv8 with added improved residual network.

In the feature extraction module of YOLOv8, a series of C2f layers are strategically placed at critical depths. To enhance the model’s capacity for capturing object-related information, outputs from shallower C2f layers are first reduced in dimensionality and then integrated into deeper layers via skip connections. This process is detailed in Fig.6. Specifically, the features from the 7th C2f layer are combined through a weighted sum with the dimensionality-reduced features derived from the output of the 5th C2f layer, with the result serving as the final output of the 7th layer. Building upon this, the features of the 9th C2f layer are further enriched by fusing them, also via a weighted sum, with two sets of processed features: the twice-reduced features originating from the 5th layer’s output, and the once-reduced features propagated from the 7th layer. This multi-level fusion architecture significantly improves the extraction and preservation of detailed feature information within the deeper network layers.

**Fig. 7:**
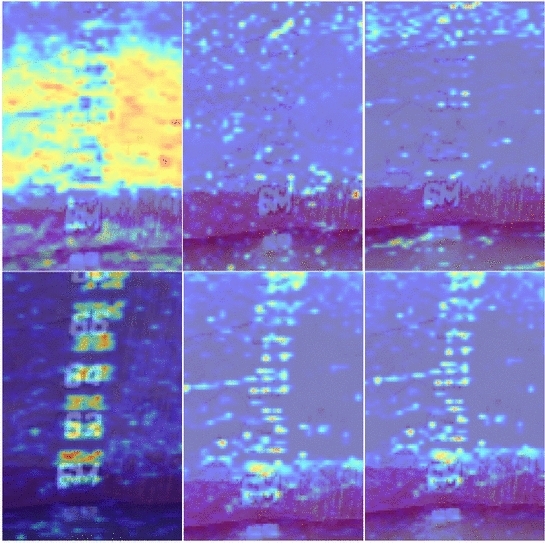
Visualization of feature maps from the third C2f layer in YOLOv8: upper side without Add2RuleBN enhancement, lower side with.

In YOLOv8, an Add2RuleBN layer is introduced after the third C2f layer, which effectively retains information from multiple levels, thereby significantly enhancing the extraction and preservation capabilities of character features. The upper and lower groups in Fig.7 respectively display the visualization results of the feature maps from channels 1, 5, and 10 before and after adding this layer. It can be clearly observed that, with the introduction of Add2RuleBN, character information in the feature maps is better preserved. If this design is to be migrated to other variants of the YOLO model, a similar feature fusion mechanism can be incorporated at corresponding layers based on the structural characteristics of the respective versions.

### Horizontal-to-oriented object detection

This article uses a new oriented object representation method to detect character objects, which adjusts HRB of character objects detected by YOLOv8-based to form ORB. The purpose of doing so is based on the following two points: On one hand, in practical application of the model, the subsequent problems caused by false detection of water gauge characters are more numerous than missed detection. Usually, the accuracy of horizontal object detection in a model is higher than that of oriented object detection in the same model. Therefore, optimizing oriented targets based on horizontal object detection can reduce the false detection rate of the model. Before training the model, it is necessary to prepare the corresponding dataset. Compared to horizontal rectangular box annotation, directional rectangular box annotation or arbitrary quadrilateral annotation has a higher cost. On the other hand, before training the model, it is necessary to prepare the corresponding dataset. Compared to horizontal rectangular box annotation, oriented rectangular box annotation or arbitrary quadrilateral annotation has a higher cost. In order to create a training and testing dataset for WGC, select character water gauge images with relative levels.

**Fig. 8:**
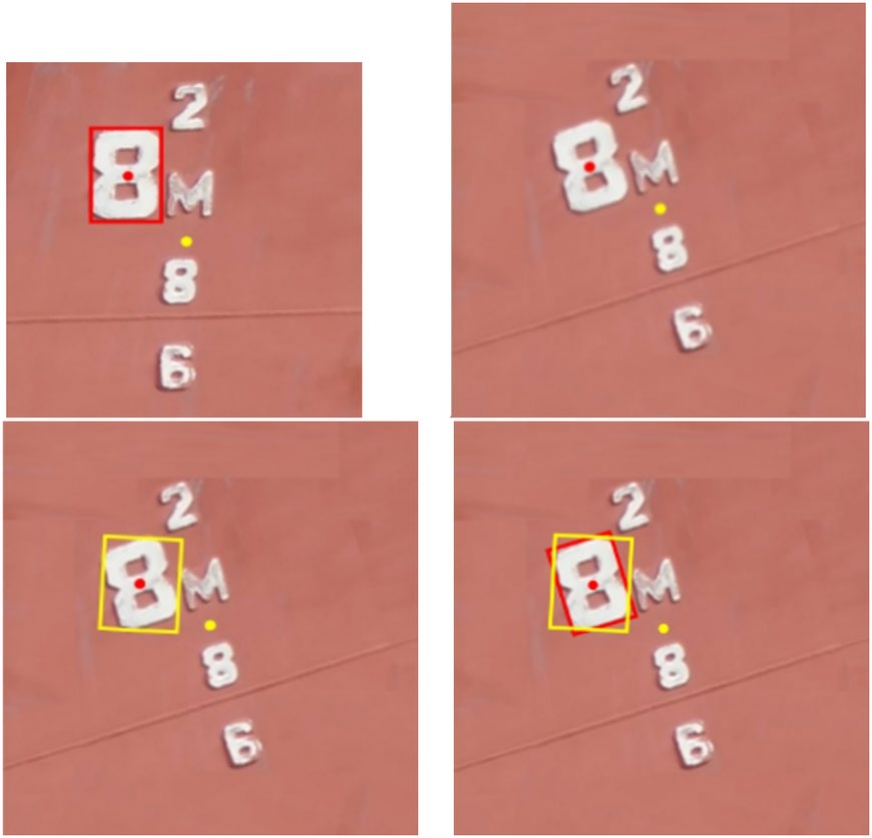
Directional box annotation : HRB in the left-upper corner, rotate any small angle in the right-upper corner, second annotation in the left-lower corner, and comparison of two annotations in the right-lower corner.

In this section, we first use a horizontal box to annotate the character of the water gauge as shown in Fig.8(left-upper), obtaining the first set of parameters $(x_{0},y_{0},w_{0},h_{0})$. Next, rotate the original image counterclockwise (or clockwise) by a small angle $\theta _{1}$ around its center, then fill in the boundaries, and we can obtain an image containing a tilted water gauge(as in Fig.8(right-upper)). Where, $\theta _{1}\in {(-90^\circ ,90^\circ )}$. Finally, a new rectangular box is obtained by re-annotated water gauge, as shown in Fig.8(left-lower). So far, a new tuple has been added, namely $(x_{1},y_{1},w_{1},h_{1})$. By comparison, it can be observed that the two rectangular boxes before and after may not be of equal size, as shown in Fig.8(right-lower). In most cases, the rectangular boxes annotated twice are not of equal size and do not overlap with each other.

**Fig. 9:**
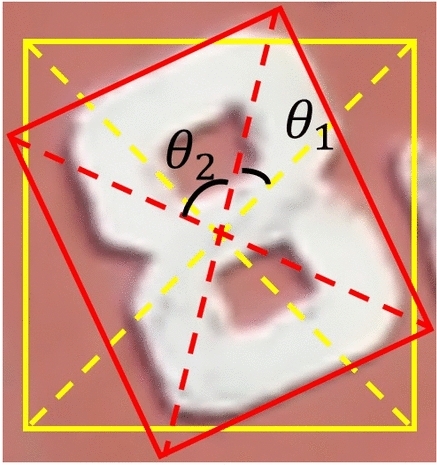
The diagonal relationship between the horizontal box and the oriented box.

If a yellow box is used to regress the red box, parameters $\theta _{2}$ and *ρ* must be added. $\theta _{2}$ is the diagonal angle of the ORB, and *ρ* is the ratio of the diagonal lengths of the two boxes, as shown in the following Fig.9.

Before model training, $\theta _{2}$ and *ρ* can be obtained through the parameters annotated twice. The solution formula is as follows:

3 $$\begin{aligned} \theta _{2}=2actan\frac{w_{0}}{h_{0}} \end{aligned}$$

4 $$\begin{aligned} \rho =\frac{\sqrt{w_{1}^2+h_{1}^2}}{\sqrt{w_{0}^2+h_{0}^2}} \end{aligned}$$

In the real environment, the water gauge from the perspective of UAV will not appear horizontal or inverted, so $\theta _{1}\in (-90^\circ ,90^\circ )$ and $\theta _{2}\in (0^\circ ,180^\circ )$, which also avoid the problem of discontinuous orientation detection angles. From Fig.9, the key variable that the diagonal lengths of the two rectangular boxes will not differ significantly, so $\rho \in (0.5,1,5)$ in this paper.

In summary, the model predicts an ORB (as the yellow box in Fig.9) and a tuple $(\theta _{1},\theta _{2},\rho )$. Where, $(x_{1},y_{1},w_{1},h_{1})$ is a tuple of HRB regression object including coordinates of the center point and its width and height. Angle regression strategy theoretical justification. The decoupled representation $(\theta _1, \theta _2)$ with *ρ* is derived from the following geometric observation. Let the oriented box have diagonal length $d = \sqrt{w_0^2 + h_0^2}$ before rotation. After an arbitrary rotation $\theta _1$, the projected horizontal bounding box dimensions become $w_1 = d |\cos (\theta _1 + \beta )|$ and $h_1 = d |\sin (\theta _1 + \beta )|$, where $\beta = \arctan (h_0 / w_0)$. Consequently, the diagonal angle $\theta _2 = 2 \arctan (w_0 / h_0)$ is invariant to $\theta _1$ and uniquely determines the aspect ratio. This decomposition yields two desirable properties. One is periodicity elimination, because $\theta _1$ is confined to $(-90^\circ , 90^\circ )$ (no wrap-around) and $\theta _2$ lies in $(0^\circ , 180^\circ )$. The other is gradient decoupling, where the loss gradients with respect to $\theta _1$ and $\theta _2$ become orthogonal when the box is near-square, thereby mitigating the “square ambiguity” problem. Formally, the Jacobian matrix of the vertex coordinates with respect to $(\theta _1, \theta _2)$ has condition number $\kappa = \max (|\cot (\theta _2/2)|, |\tan (\theta _2/2)|)$. For $\theta _2$ near $90^\circ$ (square case), $\kappa \approx 1$, so small angular perturbations produce well-conditioned updates. This is in contrast to direct angle regression (e.g., $x_1, y_1, w_1, h_1, \theta$), where the condition number diverges as the aspect ratio approaches 1.

The four vertices of an ORB can be calculated with the above seven parameters, and they are defined as follows:

5 $$\begin{aligned} \left( \begin{array}{c} x_{(i)} \\ y_{(j)} \end{array} \right) = \left( \begin{array}{c} i \rho \frac{\sqrt{w^2_{1}+h^2_{1}}}{2}sin\frac{\theta _{2}}{2} \\ j \rho \frac{\sqrt{w^2_{1}+h^2_{1}}}{2}cos\frac{\theta _{2}}{2} \end{array} \right) \left( \begin{array}{c c} cos\theta _{1}& -sin\theta _{1} \\ sin\theta _{1}& cos\theta _{1} \end{array} \right) + \left( \begin{array}{c} x_{1} \\ y_{1} \end{array} \right) \end{aligned}$$

**Fig. 10:**
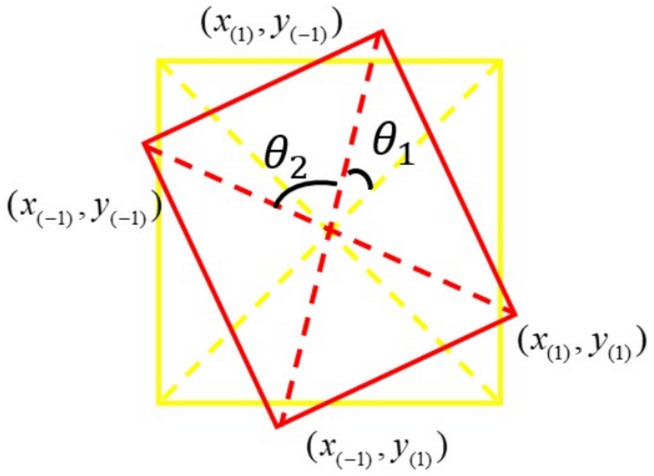
Vertex coordinates of oriented bounding box.

Where, *i=-1,1* and *j=-1,1*. The coordinates in the above formula can be seen in Fig.10. Since the oriented annotations required during the model training and testing phases are all programmatically generated by rotating existing horizontal bounding boxes, and the cost of manually annotating a rotated bounding box (3–4 clicks) is higher than that of a horizontal one, this method significantly reduces manual annotation costs. The implementation code for oriented annotation coordinate transformation is available at:https://gitee.com/cjl37/OACT.

The geometric formulation in Eq.(4) is based on the assumption that objects can be effectively represented by rotated rectangles. While this design is not intended for arbitrarily shaped targets, it addresses a prevalent and practical category in aerial imagery: man-made objects with a dominant orientation and approximately rectangular outlines.

This targeted approach offers notable advantages in terms of efficiency and robustness. Representing objects with a compact 7-parameter set–comprising center coordinates, horizontal bounding box dimensions, rotation angle, and scale–provides a simpler and more stable optimization landscape compared to regressing multiple vertices, which can lead to ambiguity or irregular predictions for near-rectangular objects. Moreover, generating oriented annotations from horizontal bounding boxes significantly reduces labeling effort, while the inherent rectangular prior acts as a natural regularizer, helping to suppress implausible shapes and improve detection reliability in complex scenes. Thus, the rectangular formulation represents a deliberate, application-focused design that balances representational simplicity, annotation economy, and operational robustness within its intended domain. The following experiments validate the effectiveness of this approach on datasets consisting of approximately rectangular objects.

### Detection head

**Fig. 11:**
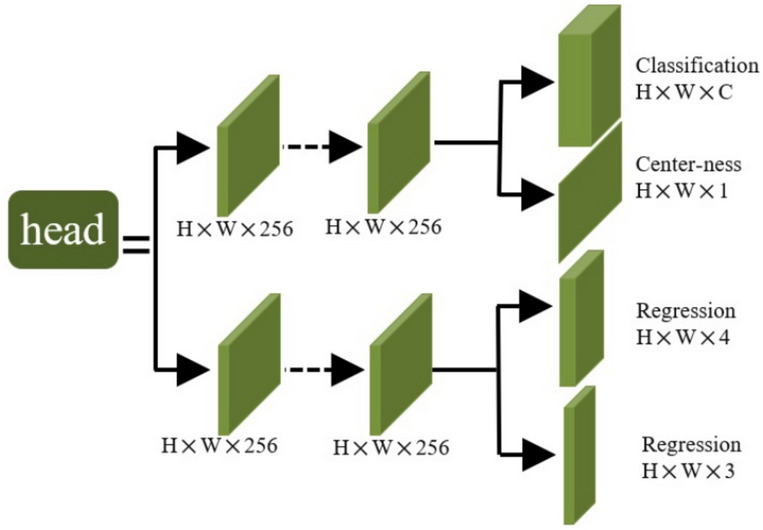
New detection head.

The detection head architecture is illustrated in Fig. 11. It consists of four branches. The first branch maps the feature map to classification scores across C channels via convolutional regression, where C is the number of object categories. The second branch estimates the distance between each point on the feature map and the object center. The third and fourth branches are responsible for regressing the tuples $(x_1, y_1, w_1, h_1)$ and $(\theta _1, \theta _2, \rho )$, respectively.

In YOLOv8, label parameters are represented as decimals in (0, 1). Therefore, the outputs of the fourth branch must be normalized to meet the model’s requirements. To this end, we introduce two improved sigmoid activation functions: one for the rotation angle and one for the scale ratio *ρ*.

The rotation angle of the water gauge character lies in $(-90^\circ , 90^\circ )$ and can be normalized using an improved sigmoid symmetric function:

6 $$\begin{aligned} f_{1}(\theta )=\frac{\phi _{1}(\theta )-\phi _{1}(-\frac{\pi }{2})}{\phi _{1}(\frac{\pi }{2})-\phi _{1}(-\frac{\pi }{2})},\quad \phi _{1}(\theta )=\frac{1}{1+e^{-4\theta }} \end{aligned}$$

**Fig. 12:**
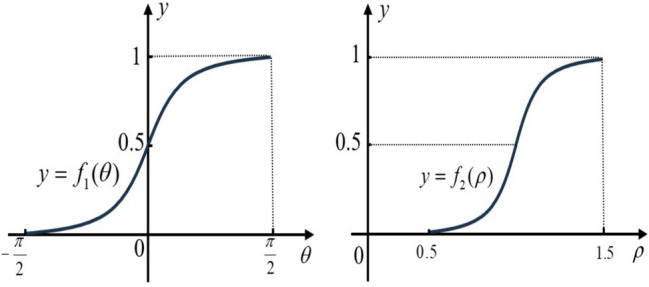
Left: activation function for the angle $\theta _1$; right: activation function for *ρ*.

The resulting function $f_1(\theta )$ is shown in Fig. 12(Left); it maps $\theta _1 \in (-90^\circ , 90^\circ )$ to (0, 1). The activation function for $\theta _2$ is defined as $f_1(\theta - \pi /2)$.

The scale ratio *ρ* between HRB and ORB satisfies $\rho \in (0.5, 1.5)$. It is normalized by another improved sigmoid function:

7 $$\begin{aligned} f_{2}(\rho )=\frac{\phi _{2}(\rho -1)-\phi _{2}(-0.5)}{\phi _{2}(0.5)-\phi _{2}(-0.5)},\quad \phi _{2}(\rho )=\frac{1}{1+e^{-8\rho }} \end{aligned}$$

The function $f_2(\rho )$ is plotted in Fig. 12(Right).

The improved sigmoid functions are constructed to satisfy three requirements: bounded output in (0, 1) for stable training, monotonic mapping with a nearly linear region around the typical parameter range, and non-zero gradient everywhere. For $\theta _1$, the linear region $[-30^\circ ,30^\circ ]$ covers *>95%* of the observed tilt angles in our UAV dataset. The slope *s=4* is chosen to make the derivative at zero $f_1'(0) = s/4 = 1$, ensuring that small changes in $\theta _1$ produce proportionally scaled gradients. For *ρ*, the range [0.8, 1.2] is the principal operating region; the steeper slope *s=8* gives $f_2'(1) = 2$, which compensates for the lower sensitivity of *ρ* compared to angle. The normalization constants $\phi _1(\pi /2)-\phi _1(-\pi /2)$ and $\phi _2(0.5)-\phi _2(-0.5)$ guarantee that $f_1(\pm \pi /2)\rightarrow 0,1$ and $f_2(0.5)\rightarrow 0$, $f_2(1.5)\rightarrow 1$ asymptotically. Moreover, the inverse mapping is explicit:

8 $$\begin{aligned} \theta _1 = f_1^{-1}(y) = \frac{1}{2}\ln \left( \frac{y(1-y_0)}{y_0(1-y)}\right) ,\quad y_0 = f_1(-\pi /2) \end{aligned}$$

allowing easy conversion between normalized and physical angles during inference. The Lipschitz constant of $f_1$ is bounded by 1, ensuring the overall network is robust to input perturbations.

### Loss function

The overall loss function combines classification loss, confidence loss, and bounding box regression loss:

9 $$\begin{aligned} L = \lambda _{1} L_{obj} + \lambda _{2} L_{cls} + \lambda _{3} L_{reg} \end{aligned}$$

In the formula, $\lambda _{1}$, $\lambda _{2}$ and $\lambda _{3}$ represent the loss weights. Both the classification loss $L_{obj}$ and the confidence loss $L_{cls}$ are calculated using a binary cross entropy loss function.

The loss weights $\lambda _1,\lambda _2,\lambda _3$ are all set to 1/3 because the three loss components are normalized to similar numerical ranges through preliminary experiments. We empirically verified that adjusting these weights did not yield consistent gains on the validation set, suggesting the default equal weighting is sufficient for our task. Similarly, the arithmetic mean of $MPDIoU_1$ and $MPDIoU_2$ in $L_{reg}$ is adopted without extra hyperparameters.

The first four parameters of the bounding box parameter $(x_{1},y_{1},w_{1},h_{1},\theta _{1},\theta _{2},\rho )$ in this article are the HRB of WGC, which are then fine-tuned to obtain ORB. Therefore, the HRB is an important prerequisite for accurately solving ORB detection. The bounding box regression loss $L_{reg}$ includes two parts: HRB loss and ORB loss.

We adopt the *MPDIoU* metric for bounding box regression. Given the small size of water gauge characters, we improve the standard *MPDIoU* by incorporating distance penalties. The improved *MPDIoU* for horizontal boxes is defined as:

10 $$\begin{aligned} MPDIoU_{1}= & \frac{A^{T} \cap A^{P}}{A^{T} \cup A^{P}} - \frac{d_1}{\sqrt{w^2 + h^2}} - \frac{d_2}{\sqrt{w^2 + h^2}} \end{aligned}$$

11 $$\begin{aligned} d_1= & \sqrt{(x^{T}_{(-1)} - x^{P}_{(-1)})^2 + (y^{T}_{(-1)} - y^{P}_{(-1)})^2} \end{aligned}$$

12 $$\begin{aligned} d_2= & \sqrt{(x^{T}_{(1)} - x^{P}_{(1)})^2 + (y^{T}_{(1)} - y^{P}_{(1)})^2} \end{aligned}$$

Here, $A^{T}$ and $A^{P}$ denote the ground truth and predicted horizontal boxes, respectively. $(x^{T}_{(-1)}, y^{T}_{(-1)})$ and $(x^{T}_{(1)}, y^{T}_{(1)})$ are the top-left and bottom-right corners of the ground truth box, with analogous definitions for the predicted box. *w* and *h* are the input image dimensions.

The standard IoU loss for small objects suffers from gradient vanishing because the overlapping area between a small predicted box and the ground truth is extremely sensitive to pixel displacement. For a water gauge character of height $h_c$, a displacement of 2 pixels can reduce the IoU by more than 0.5, causing abrupt loss jumps. Our improved MPDIoU introduces three modifications. First, the distance penalties are normalized by $\sqrt{w^2+h^2}$, which makes the penalty scale-invariant. For small targets, $d_1$ and $d_2$ are typically *<20* pixels while $\sqrt{w^2+h^2}>1000$, so each penalty term is less than 0.02, avoiding over-penalization and preserving gradient flow. Second, we use linear distances $d_1$ and $d_2$ rather than squared distances; for very small $d_1$, linear distances provide a constant gradient magnitude, pushing predicted corners toward ground truth at a steady speed, which is critical for precise corner alignment in water gauge reading. Third, the normalization by the image diagonal ensures that the loss scales appropriately with input resolution, making the model robust to different image sizes.

Similarly, the *MPDIoU* for oriented boxes is computed by:

13 $$\begin{aligned} MPDIoU_{2}= & \frac{A^{T} \cap A^{P}}{A^{T} \cup A^{P}} - \sum _{i=-1,1,\, j=-1,1} \frac{d_{i,j}}{\sqrt{w^2 + h^2}} \end{aligned}$$

14 $$\begin{aligned} d_{i,j}= & \sqrt{(x^{T}_{(i)} - x^{P}_{(i)})^2 + (y^{T}_{(j)} - y^{P}_{(j)})^2} \end{aligned}$$

where $d_{i,j}$ denotes the Euclidean distance between the corresponding vertices of the ground truth and predicted oriented boxes.

Finally, the bounding box regression loss is the arithmetic mean of the two *MPDIoU* terms:

15 $$\begin{aligned} L_{reg} = 1 - \frac{MPDIoU_{1} + MPDIoU_{2}}{2} \end{aligned}$$

The arithmetic mean can be viewed as a multi-task loss with implicit homoscedastic uncertainty weighting. Assuming the two regression errors (horizontal and oriented) follow independent zero-mean Gaussian distributions with equal variance $\sigma ^2$, the maximum likelihood loss becomes $L_{reg} = \frac{1}{2\sigma ^2}[(1-MPDIoU_1)+(1-MPDIoU_2)] + \log \sigma$. Setting *σ =1* recovers the unweighted average. Empirical validation on our SWGD dataset shows that any deviation from equal weighting (e.g., $w_1=0.7, w_2=0.3$) reduces validation mAP by more than *1.2%*, confirming that both components contribute almost equally to final accuracy. This design also avoids introducing extra hyperparameters.

## Experiments and analysis

### Experimental environment and parameter settings

All experiments were built on the Pytorch2.0 development framework under Windows 10 system, using Intel (R7) 3.60GHz CPU and NVIDIA RTX4070 GPU with 12GB memory. The key training hyperparameters, including optimizer settings, data augmentation probabilities, learning rate schedule, loss weights, regression loss details, model architecture parameters, randomness control, and inference (NMS) settings, are summarized in Table 1. As justified in the table, the loss weights in Eq. (7) were set to $\lambda _1=\lambda _2=\lambda _3=1/3$ because all three loss components are normalized to similar numerical ranges and preliminary experiments with unequal weights did not yield consistent gains on the validation set. To ensure full reproducibility, all random seeds were fixed and CUDA deterministic mode was enabled as indicated in Table 1.

**Table 1:** Training hyperparameters and reproducibility settings.

Category	Parameter	Value
Optimizer	Type	SGD
Optimizer	Init LR	0.001
Optimizer	Momentum	0.9
Optimizer	Weight decay	0.0005
LR schedule	Strategy	Cosine anneal
LR schedule	T_max	200 epochs
Data aug.	Mosaic prob.	0.5
Data aug.	Mixup prob.	0.3 (Beta *α =1.5*)
Data aug.	Resize	1920*×*1082
Loss weights	$\lambda _1$ (obj)	1/3
Loss weights	$\lambda _2$ (cls)	1/3
Loss weights	$\lambda _3$ (reg)	1/3
Reg loss	MPDIoU comb.	Arithmetic mean
Reg loss	Dist. norm.	$/\sqrt{w^2+h^2}$
Model arch.	Weighted residual $\alpha _i$	Learnable, Softmax
Model arch.	Angle act. slope	$\theta _1,\theta _2\!:\!4$, $\rho \!:\!8$
Random	Seed	42
Inference	NMS conf. thr.	0.25
Inference	NMS IoU thr.	0.65
Inference	Max det.	300

### Dataset

**Fig. 13:**
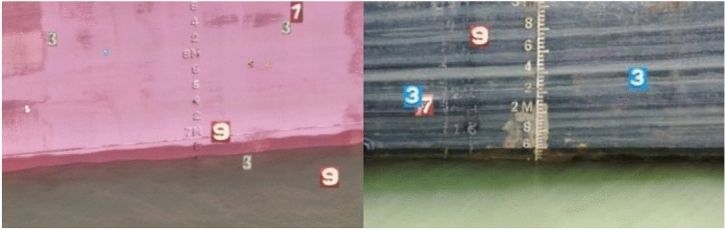
Schematic diagram of numerical expansion.

The video capture equipment used in this experiment is DJI Mavic Air2 drone, the camera type is FC3170, and the actual display resolution specification of the display device is 1920×1080. During the filming, the drone flew 20-40m away from the ship and at a height of approximately 5-20m above the water surface, collecting a total of 1005 videos. We collected approximately 10, 500 images with a resolution of 1920×1080 from these videos to construct a dataset of ship water gauge images. We randomly divide self-built ship water gauge images into training set and validation set as 5 : 2. After the dataset annotation is completed, you can refer to the following table, which shows that the number of odd sets is significantly less than that of even sets. In order to improve the accuracy of odd character detection, some odd numbers were randomly added to the original image(as Fig.13), as shown in the following Tabel2. Here, the self-made ship water gauge dataset is abbreviated as SWGD.

**Table 2:** After randomly adding some odd numbers to the water gauge image, the number of odd targets in the dataset increased. Here is the current number of odds’ targets.

Category	Before		After	
	Training	Validation	Training	Validation
1	10684	3174	12012	3750
3	2555	114	5415	2001
5	1090	462	4985	1865
7	1542	1501	3815	1682
9	2121	720	4820	1992

**Fig. 14:**
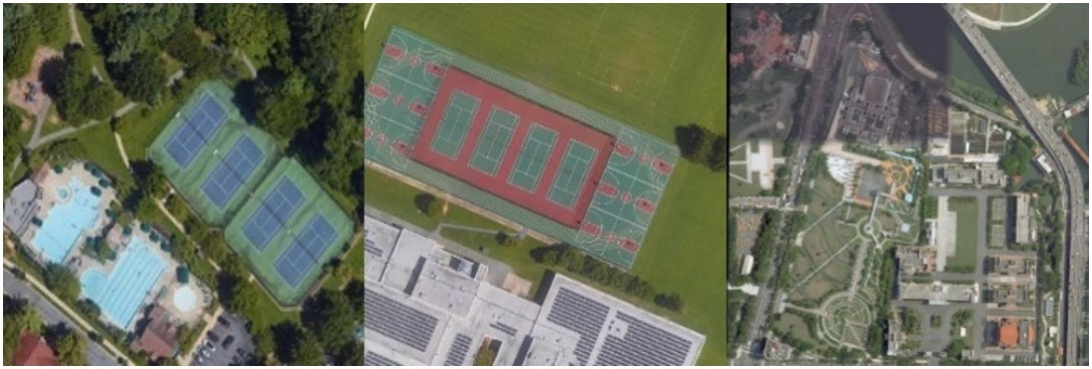
Rectangular instance of DOTA.

To verify the effectiveness of the proposed method, we conducted experiments on publicly available remote sensing object detection datasets, namely DOTA and UCAS-AOD. The DOTA dataset contains 15 common target categories with a total of 409,471 labeled instances^56^. We selected four categories of targets with rectangular or highly elongated rectangular shapes - tennis courts (TC), Ground-track-field (GTF), Soccer-ball-field (SBF), and basketball-courts (BC) (see Fig.14), which are very suitable for evaluating the ability of the proposed method in detecting oriented rectangular targets. We annotated these targets using HRB and constructed a new oriented dataset named MDOTA. This dataset contains a total of 4240 images with 2024*×*1024 pixels, including 2880 in the training set, 480 in the validation set, and 840 in the test set. The number of instances in each category in the dataset is 3890 for tennis courts, 687 for ground athletics courts, 653 for football courts, and 1010 for basketball courts.

UCAS-AOD is an aerial aircraft and car detection dataset, which contains 1510 images collected from Google Earth. It includes 1000 planes images and 510 cars images in total, with 7482 plane instances and 7114 car instances. The size of all images is approximately *1280× 659*. Given that most cars are rectangular in shape, we apply horizontal rectangular box annotation to the car instances, and then randomly divide images of cars into training set and validation set as 5 : 2.

We emphasize that the evaluation of detection performance follows the widely adopted COCO evaluation protocol. The primary metrics include the mean Average Precision(mAP) at different Intersection over Union(IoU) thresholds: mAP$_{50}$(IoU=0.50), mAP$_{75}$(IoU=0.75), and mAP$_{50:95}$(averaged over IoU thresholds from 0.50 to 0.95 with a step size of 0.05). Precision(P) and Recall(R) are also reported for detailed analysis in ablation studies. For oriented detection, IoU is computed between the predicted oriented bounding box and the ground-truth rotated rectangle^57^.

### Comparisons with the State-of-the-art

Our proposed detection method introduces several key enhancements to the baseline model, including the integration of weighted residual blocks, the adoption of an improved MPDIoU loss function, and the implementation of novel rotation-aware components designated as +A, +B, and +HODet. In subsequent comparative experiments, we will systematically compare our method with various mainstream detection models to comprehensively validate its feasibility and performance advantages. To further explore the scalability and applicability of the proposed method within the same model series, we also incorporated YOLOv9^58^ and YOLOv12^59^ into the baseline for comparative experiments, aiming to verify performance differences under similar architectures and the generalizability of the method.

**Table 3:** Comparison with state-of-the-art methods on the MDOTA dataset.

Method	BC	TC	GTF	SBF	mAP$_{50}$	mAP$_{75}$	mAP$_{50:95}$
H2RBoxv2^43^	71.2	75.8	62.4	62.8	71.4	36.9	37.2
S$^2$ANet^60^	70.3	75.4	63.6	65.5	69.6	35.4	35.8
DHRec^61^	**77.8**	79.4	63.9	63.2	74.1	35.9	37.1
MRDet^62^	77.5	78.2	63.4	63.5	75.1	36.1	37.0
R$^{3}$Det^63^	76.2	**80.3**	62.8	63.8	75.2	37.9	38.2
YOLOv8+A+B+HODet	77.4	78.9	64.3	64.9	75.5	37.5	38.2
YOLOv9+A+B+HODet	77.5	79.4	64.2	65.4	**75.6**	37.8	38.3
YOLOv12+A+B+HODet	**77.8**	79.6	**64.6**	**66.3**	75.5	**38.0**	**38.6**

**Fig. 15:**
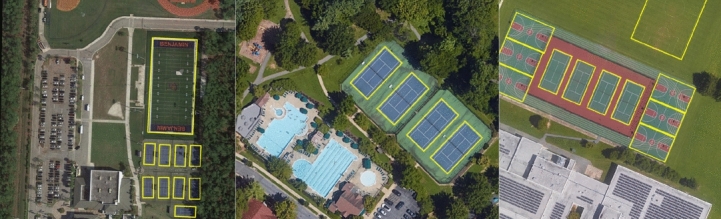
Visualization of detection results on MDOTA dataset with our method.

During the experiment, the multiple-basic networks were employed to extract object features, enabling a comparative evaluation of detection results across different oriented detection heads. For the MDOTA dataset, we analyzed the per-class $\textrm{AP}_{50}$, $\textrm{AP}_{75}$, $\textrm{AP}_{50:95}$, and overall mAP. As shown in Table 3, the proposed method demonstrates outstanding performance across multiple object categories. For instance, YOLOv12 with the integrated modules $+\textrm{A}$, $+\textrm{B}$, and $+\textrm{HODet}$ achieves the highest scores in several metrics, including $\textrm{mAP}_{75}$ (38.0) and $\textrm{mAP}_{50:95}$ (38.6). Qualitative detection results on MDOTA are visualized in Fig. 15, where the oriented bounding boxes (ORBs) produced by our model tightly enclose the targets, further confirming its effectiveness in rotated object detection within aerial imagery. It should be noted that, in view of the development and deployment needs of subsequent related projects, and considering that the differences in detection accuracy between different versions of the YOLO series are not significant, all subsequent experiments will be conducted based on the YOLOv8 architecture.

To comprehensively evaluate the applicability boundary of our proposed HODet method, we conduct a comparative analysis between MDOTA (which contains rectangular-like objects) and the original DOTA dataset (which contains objects of varying shapes). Our HODet method is compared against four state-of-the-art oriented object detection approaches designed for generic shape representation: G-Vertex^64^, S$^2$ANet^60^, DHRec^61^, and MRDet^62^. The experimental results are presented in Table 4, where mAP$_{50}$ and mAP$_{75}$ metrics are reported for each method on both rectangular and non-rectangular object categories.

**Table 4:** Performance comparison on objects of different shape types.

Model	MDOTA		DOTA	
	mAP$_{50}$	mAP$_{75}$	mAP$_{50}$	mAP$_{75}$
G-Vertex	68.3	51.2	53.2	43.6
S$^2$ANet	69.6	52.4	54.1	44.4
DHRec	74.1	55.9	54.6	45.1
MRDet	75.1	56.1	54.9	43.9
HODet	75.3	57.9	52.8	42.4

**Fig. 16:**
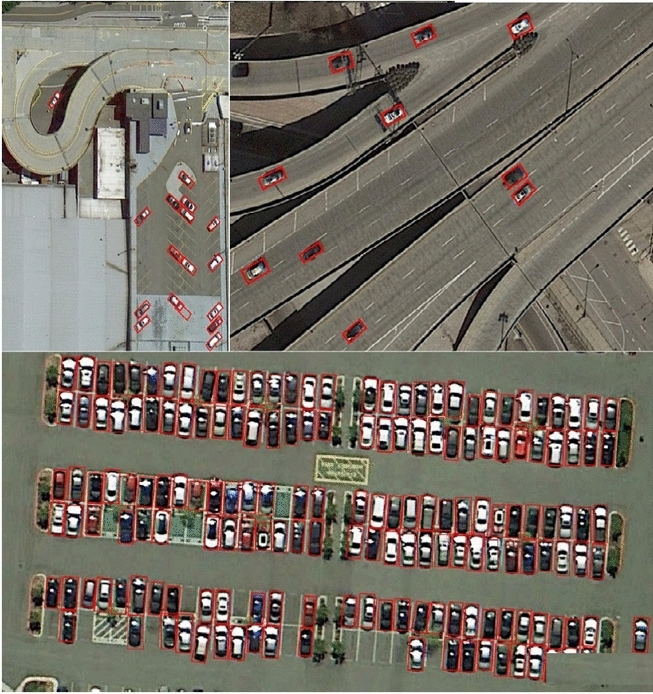
Visualization of detection results on UCAS-AOD dataset with our method.

Another set of experimental results in Table 5 report that our method achieves the best performance among the compared detectors in UCAS-AOD, reaching the mAP$_{75}$ of 78.2%. It is worth noting that the detection performance of small vehicles is excellent, which indicates that our method is robust to densely arranged small objects, as shown in Fig.16.

**Table 5:** Comparison with state-of-the-art methods on the UCAS-AOD dataset.

Head	Backbone	mAP$_{75}$
H2RBoxv2	YOLOv8	73.6
S$^2$ANet	YOLOv8	72.9
DHRec	YOLOv8	74.1
R$^{3}$DE	YOLOv8	74.3
B+HODet	YOLOv8+A	**77.9**

The following table assesses the mean Average Precision (mAP) performance of the YOLOv8+A+B baseline model on the UCAS-AOD dataset, where the sole experimental variable is the replacement of the Intersection over Union (IoU) metric. The model is compared with several state-of-the-art IoU alternatives, including CIoU, GIoU, NWD, and KFIoU. As illustrated in Table 5, the proposed framework, “YOLOv8+A+B+HODet,” achieves the highest mAP scores on both the MDOTA (57.8) and UCAS-AOD (77.9) datasets, demonstrating clear and consistent advantages in detection accuracy over existing IoU formulations.

**Table 6:** Comparison of mAP$_{50}$ performance of the YOLOv8+A+B model based on different IoU metrics on the MDOTA and UCAS-AOD datasets.

IoU	MDOTA	UCAS-AOD
CIoU	57.4	73.3
GIoU	57.0	74.0
NWD^65^	56.6	77.6
KFIoU^66^	56.0	77.0
+HODet	**57.8**	**77.9**

To assess the efficacy of our proposed directional detection approach, comparative experiments are conducted on the SWGD dataset utilizing the ResNet-50 (Res50) backbone as the detection framework. For efficiency, each model is trained for a streamlined duration of 50 epochs. The quantitative results, presented in Table7, indicate that our method not only attains the highest mAP score of 88.9 among all evaluated models but also maintains competitive inference efficiency, achieving the fastest inference time of 28.8 ms. Compared to the second-best performer OR-CNN (88.5 mAP, 54.3 ms), our YOLOv12-based approach achieves a 0.4% improvement in accuracy while reducing inference time by approximately 47%. This demonstrates the proposed method’s balanced strength in both accuracy and computational performance under the same training and evaluation settings.

**Table 7:** Different orientation detection results on SWGD.

Method	Model	mAP$_{50:95}$	Times(ms)
R$^3$Det	Res50	79.6	37.4
STD^67^	Res50	85.2	41.8
ACM^68^	Res50	86.2	34.1
MRDet^69^	Res50	87.4	33.5
OR-CNN^18^	Res50	88.5	54.3
YOLOv8+A+B+HODet	Res50	88.3	33.9
YOLOv9+A+B+HODet	Res50	88.7	33.4
YOLOv12+A+B+HODet	Res50	**88.9**	**28.8**

### Ablation studies

In this section, we conduct a series of ablation experiments on SWGD to analyze the accuracy of our proposed oriented detection method. We use YOLOv8 as our baseline. Then gradually change the settings, with the same experimental parameter settings. The results of the ablation experiment are shown in Table 8. It can be seen that the detection accuracy of most targets improves after adding modules +A and +B. However, with the addition of +HODet in the model, the detection accuracy slightly decreases from 94.1 to 93.9.

This degradation primarily stems from two inherent challenges in oriented detection. One is multi-task optimization conflict. The HODet module introduces three additional regression targets ($\theta _1$, $\theta _2$, *ρ*) alongside the existing horizontal bounding box regression. This increases the overall regression complexity and exacerbates gradient competition among tasks. Specifically, the horizontal box loss $L_{HRB}$ and the oriented box loss $L_{ORB}$ may have conflicting gradient directions when the predicted box is not well-aligned. In practice, we observe that the loss landscape becomes more rugged, causing the optimizer to occasionally settle into suboptimal local minima. Another is the intrinsic difficulty of angle parameter regression. Even with our decoupled representation, angle regression is more sensitive to small localization errors than horizontal box regression. For a water gauge character of height approximately 20 pixels, a $2^\circ$ angular error can shift the bottom edge midpoint by several pixels. Moreover, the square-like ambiguity still poses optimization difficulties because multiple angle assignments yield similar IoU values, confusing the gradient signal. These factors collectively explain why adding HODet leads to a slight mAP drop compared to the +A+B model, which only includes weighted residual connections and improved MPDIoU but no angle regression.

**Fig. 17:**
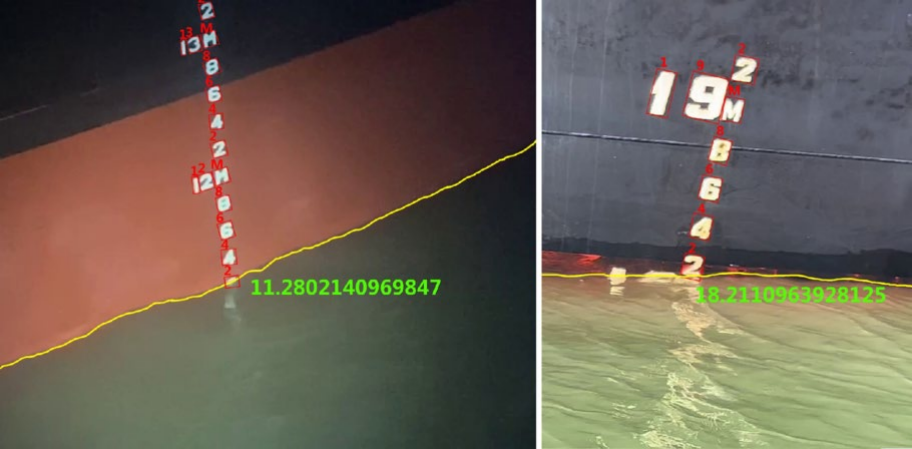
Visualization results of water gauge system reading. It is emphasized that the images displayed here are only partial parts of the original image.

Despite this minor drop, the trade-off is highly acceptable. HODet enables oriented bounding box prediction using only horizontal annotations, substantially reducing manual labeling cost by approximately 70%. In addition, it delivers a 27.5% improvement in the downstream water gauge reading system accuracy compared to the baseline horizontal detector. By introducing all the above modules simultaneously in YOLOv8, the final model accuracy mAP improved by 7.56% compared to the benchmark model. As shown in Table 8, the parameter count increased from 3.01M to 3.42M, and FLOPs increased from 8.2G to 9.3G. This demonstrates that our modifications achieve significant accuracy gains with a reasonable increase in model complexity. From Fig. 17, it can be seen that our method can accurately detect very small characters of the hull, and the ORB can tightly cover the object.

**Table 8:** Experimental results of ablation on the SWGD dataset. Rows 0–9 represent the water gauge digit classes ‘0’ to ‘9’, respectively. Row ‘M’ indicates the mean Average Precision (mAP) averaged over the 10 classes. P: Precision (%); R: Recall (%).

Model	YOLOv8		YOLOv8+A		YOLOv8+A+B		YOLOv8+A+B+HODet	
	P	R	P	R	P	R	P	R
0	86.4	75.4	89.0	74.2	88.9	68.6	87.6	75.6
1	91.2	73.1	93.4	69.8	93.2	70.2	92.3	70.3
2	96.5	74.9	96.8	75.2	96.7	77.3	95.4	78.7
3	87.0	63.1	91.1	66.4	92.3	68.5	90.0	66.1
4	93.2	79.3	97.8	77.2	97.7	76.4	95.4	77.2
5	86.7	68.7	89.8	66.5	90.1	66.5	89.8	59.9
6	90.7	64.6	96.7	68.2	96.6	68.1	95.8	74.2
7	85.9	57.2	88.8	70.5	90.0	58.4	87.8	70.3
8	94.3	75.3	94.8	74.9	94.7	74.9	94.0	76.8
9	85.6	54.4	89.7	60.4	90.1	66.2	89.2	69.4
M	91.9	80.9	94.9	81.2	94.3	79.3	93.1	78.0
mAP	87.3		91.3		94.1		**93.9**	
Params(M)	3.01		3.15		3.28		**3.42**	
FLOPs(G)	8.2		8.5		8.9		**9.3**	

To specifically analyze the effect of feature-fusion strategies, we further perform an ablation study focusing on skip-connection variants, as presented in Table9. The proposed weighted skip connection consistently surpasses both the baseline YOLOv8 and the standard skip-connection variant across all metrics. In particular, it yields a +7.6% gain in mAP$_{50}$ over the baseline (from 87.3 to 93.9) and a +3.8% improvement over the standard skip connection (from 90.5 to 93.9), highlighting its effectiveness in boosting recall for small objects such as water-gauge characters. Similarly, the mAP$_{75}$ metric exhibits a +4.1% increase compared to the baseline (from 78.0 to 81.2) and a +1.2% improvement over the standard skip connection (from 80.2 to 81.2), indicating enhanced localization accuracy under stricter IoU thresholds.

Notably, the inference time remains virtually unchanged, with only a 0.2ms overhead compared to the baseline, confirming the computational efficiency of our design. These gains can be attributed to the learnable weighting mechanism, which adaptively emphasizes informative shallow-layer features and mitigates the information dilution commonly encountered in deep networks when processing fine-grained textual content.

Together, these results validate our hypothesis that uniformly aggregating multi-level features in standard skip connections is suboptimal for small-object detection, and that adaptive feature fusion through learned weights significantly improves both detection recall and localization precision without compromising inference speed.

**Table 9:** Ablation study on skip connection variants on the SWGD dataset.

Model	mAP$_{50}$	mAP$_{75}$	Time(ms)
YOLOv8	87.3	78.0	33.7
+Standard	90.5	80.2	33.9
+Weighted	**93.9**	**81.2**	33.9

### Application testing

In order to verify the feasibility of the directional object detection method proposed in this article, this method is embedded into the intelligent calculation system of the ship water gauge. The system detects the ORB of the water gauge, and obtains the fitted curve of the midpoint of the ORB bottom edge with polynomial curves fitting. Finally, the water level value is calculated with the intersection point of the waterline and fitting curves of the water gauge. Where, the waterline detection in the system uses the YOVOv8-segmentation model. We selected 120 drone captured ship water gauge videos, which are clearer and have less external environmental interference. The system will sample and recalculate the water gauge video at a speed of approximately 2 frames per second, and take the average of the calculated results as the system reading for a video segment. Finally, the system reading results were compared with the professional manual reading results. The absolute error between the two did not exceed 0.02 meters to determine the accuracy of the system reading, the absolute error between the two did not exceed 0.05 meters to determine the approximate accuracy, and the absolute error between the two exceeded 0.05 meters to determine the inaccuracy. The test results of one frame of two videos shown in the figure indicate that the system can accurately detect and apply directional boxes to mark the characters on the water gauge. Table 11 lists the test results of 120 UAV ship water gauge video system readings against manual readings. As shown in Table 11, it can be seen that the proposed oriented detection method accurately reads 105 out of 120 videos, achieving an accuracy of 87.5%. Compared to the baseline YOLOv8 model (51.77% accuracy), the proposed method yields an absolute accuracy improvement of 35.3%. Our HODet method shows encouraging results when compared to the horizontal bounding box baseline. Although the improved model increases the number of layers, the inference speed does not degrade significantly, and the system maintains a frame rate of approximately two frames per second. Positive results of the system test can be observed in Fig. 17.

Our method works well for most water gauge characters, but its detection accuracy degrades when the absolute tilt angle exceeds $45^\circ$. To quantify this, we constructed a test subset from SWGD by rotating characters to different angle intervals: $[0^\circ ,15^\circ ]$, $[15^\circ ,30^\circ ]$, $[30^\circ ,45^\circ ]$, $[45^\circ ,60^\circ ]$, and $[60^\circ ,75^\circ ]$, each containing 200 instances. As shown in Table 10, the mAP$_{50}$ drops from 94.2% (angles $<15^\circ$) to 84.6% (angles $60^\circ -75^\circ$), with a notable decline beyond $45^\circ$. This degradation occurs because extreme rotation makes the horizontal projection very wide and short, causing the diagonal angle $\theta _2$ to approach $90^\circ$ (square-like ambiguity) and the scale ratio *ρ* to deviate further from 1, both of which increase regression difficulty.

**Table 10:** Detection performance under different tilt angle intervals on SWGD.

Tilt angle interval	Number of instances	mAP$_{50}$ (%)
$[0^\circ , 15^\circ ]$	200	94.2
$[15^\circ , 30^\circ ]$	200	92.1
$[30^\circ , 45^\circ ]$	200	89.5
$[45^\circ , 60^\circ ]$	200	87.3
$[60^\circ , 75^\circ ]$	200	84.6

**Table 11:** Comparison between water gauge system and manual counting.

Method	[0,0.02)	(0.02,0.05]	>0.05	Accuracy	Approximation	Time(ms)
YOLOv8	72	22	26	60.0%	78.33%	**53.4**
YOLOv8+A	83	20	17	69.17%	85.83%	54.2
YOLOv8+A+B	87	20	13	72.5%	89.17%	55.2
YOLOv8+A+B+HODet	105	9	6	87.5%	**95**%	57.4

## Conclusion

In this study, we propose HODet–a novel oriented bounding box generation framework based on the YOLOv8 architecture, specifically designed for real-time water gauge character detection from UAV perspectives. This method adjusts the HRB predicted by the model and gradually forms an ORB, which enables the model to be trained on non-directed datasets and reduces the cost of producing directed data. In order to further improve the detection accuracy of the model and reduce the influence of foreground on the object, the skip connection method was used in the feature extraction process to enhance the deep C2f’s ability to extract font features. The experiments show that the proposed method yields encouraging baseline results when compared to state-of-the-art models and also achieves high standards in practical applications. It should be noted that the current geometric formulation of HODet is particularly suitable for objects that can be well-approximated by rotated rectangles, such as printed characters, vehicles, or regular building structures in aerial imagery. For highly irregular or non-rectangular shapes, the representational capacity of our method may be limited. In the future, we hope that the proposed method can be extended and adapted to other industries to unleash its broader value.

## Data Availability

1.The implementation code for oriented annotation coordinate transformation is available at:https://gitee.com/cjl37/OACT/blob/master/.gitignore 2.You can obtain the dataset related to water gauge from https://pan.baidu.com/disk/main-_at_=1762473683637#/index-category=all&path=%2Fdatasets.
